# Strength, Weakness, Opportunities, and Threats (SWOT) Analysis of Virtual Outpatient Department Under Telemedicine Department During the COVID-19 Pandemic

**DOI:** 10.7759/cureus.22476

**Published:** 2022-02-22

**Authors:** Vartika Saxena, Yogesh Bahurupi, Ashutosh Mishra, Ashok Singh, Shailesh Parate, Harindra Sandhu

**Affiliations:** 1 Department of Community and Family Medicine, All India Institute of Medical Sciences, Rishikesh, Rishikesh, IND; 2 Department of Pathology, All India Institute of Medical Sciences, Rishikesh, Rishikesh, IND; 3 Department of Forensic Medicine, All India Institute of Medical Sciences, Rishikesh, Rishikesh, IND; 4 Department of General Surgery, Lala Lajpat Rai Memorial Medical College, Meerut, IND

**Keywords:** threats, opportunities, weakness, strength, swot analysis, virtual opd, telemedicine, covid-19

## Abstract

Background

COVID-19 pandemic has challenged all current management tools used for patient care. This study aims to determine strength, weakness, opportunities and threats (SWOT) to virtual OPD and consultants' perceptions of teleconsultation virtual OPD during the COVID-19 pandemic, adapting to newer technologies for successfully handling this situation.

Material and methods

A facility-based cross-sectional study was conducted at the Virtual OPD of All India Institute of Medical Sciences Rishikesh among patients availing Telemedicine consultation during the COVID-19 pandemic. All patients availing services from April 2020 to October 2020 were included in this study. Method for SWOT analysis: A checklist was prepared, and investigators assessed SWOT. An external evaluator was invited to evaluate the SWOT analysis conducted by the investigators. For numeric variables, the mean ± SD was used, and for categorical variables, percentages and proportions were used.

Results

Around 22% of the patients who approached virtual OPDs were ≥ 60 years of age. More than half (55.3%) of the patients or their attendants who consulted Telemedicine OPD were from Uttarakhand, followed by Uttar Pradesh (42.6%). The proportions of male patients were 54.4%. Around 17.6% of teleconsultations were performed for radiotherapy. General medicine and cardiology consultations were (15.2%) and (12.8%), respectively.

Conclusion

Telemedicine can be effective if certain requirements had been provided. Maintaining privacy of the patient's data was a challenge.

## Introduction

Telemedicine can be defined as the delivery of healthcare services, where distance is a critical factor, by all healthcare professionals using information and communication technologies for the exchange of valid information for the diagnosis, treatment, and prevention of disease and injuries, for research and evaluation, and for the continuing education of healthcare providers, all in the interests of advancing the health of individuals and their communities [1].

Due to the COVID-19 pandemic, telemedicine services were better options for patients to receive overall treatment from a distance and stay safely at home during and after the lockdown period.

Telemedicine aims to ensure equitable offerings to everyone, are cost-effective, offer protection to sufferers and medical doctors for the duration of pandemics, and provide timely and quicker care [2].

The non-accessibility of technology and communications is a key barrier in telemedicine in developing countries. According to the International Telecommunications Union data, only 31% of the developing world population uses the internet, of which 16% is used in Africa, and 90% of households do not have access to the Internet in the developing world [3].

The COVID-19 virus has created a pandemic and has challenged all current management tools used for patient care. The challenges this pandemic created are huge and have created new avenues for handling the situation. The challenge we face is providing healthcare facilities without coming into close contact with patients.

This study aims to determine “strength, weakness, opportunities, and threats (SWOT)” to the virtual outpatient department (OPD) and consultants' perceptions of teleconsultation virtual OPD during the COVID-19 pandemic, adapting to newer technologies for successfully handling this situation. We also highlight the difficulties faced in starting up services with limited resources. This study focused on patients availing teleconsultation services through virtual OPD and consultants involved in virtual OPD at All India Institute of Medical Sciences (AIIMS) Rishikesh during the COVID-19 pandemic. The purpose of this study was to assess the SWOT analysis and profile of patients who visited the AIIMS Rishikesh virtual OPD via telemedicine.

## Materials and methods

A facility-based cross-sectional study was conducted at the virtual OPD of AIIMS Rishikesh among patients availing telemedicine consultation services at AIIMS Rishikesh during the COVID-19 pandemic. All patients availing services from April to October 2020 (seven months) were included in this study. Records of patients availing teleconsultation services were accessed for their profiles. Incomplete records were excluded from the final analyses.

Method for SWOT analysis

A checklist was prepared after review of relevant literature on telemedicine practices, which included facilitating and hindering factors on telemedicine. An external evaluator was invited to conduct the SWOT analysis by the investigators.

Criteria used for external evaluators

External evaluators have worked in telemedicine/telehealth with a public health background. An invitation was sent and a checklist prepared for SWOT analysis was vetted for appropriateness and suggestions.

All the data collected during the teleconsultation period provided by patients through telephone, WhatsApp, and video calls were evaluated and reviewed.

Data analysis

Microsoft Excel 2010 was used for entering the data. EPI Info 7.0, a professional statistical tool for Windows, was used to analyze the data. For numeric variables, the mean ± SD was used, and for categorical variables, percentages and proportions were used.

## Results

In this study, a total of 5,278 patients availed teleconsultation service from virtual OPD of AIIMS Rishikesh during the COVID-19 pandemic between April 20, 2020, and October 31, 2020.

Around 22% of the patients who approached virtual OPDs were ≥ 60 years of age, and 12% of the patients were between 46 and 50 years of age. Approximately 9.6% and 9.5% of the patients were between 36 to 40 years of age and 41 to 45 years of age, respectively. The percentage of patients under five years of age was 3.8%. The proportion of males was 54.4% compared with female patients (45.6%) in the telemedicine OPD (Table 1).

**Table 1:** Age- and genderwise distribution of patients availing teleconsultation at the virtual OPD (N=5,278) OPD, outpatient department

Variable	Categories	Number (%)
Age (in years)	0 to 5	201 (3.8%)
	6 to 10	88 (1.7%)
	11 to 15	105 (2%)
	16 to 20	168 (3.2%)
	21 to 25	361 (6.8%)
	26 to 30	398 (7.5%)
	31 to 35	430 (8.2%)
	36 to 40	508 (9.6%)
	41 to 45	502 (9.5%)
	46 to 50	633 (12%)
	51 to 55	476 (9.1%)
	56 to 59	245 (4.6%)
	≥ 60	1163 (22%)
Gender	Male	2870 (54.4%)
	Female	2408 (45.6%)

More than half (55.3%) of the patients or their attendants who consulted telemedicine OPD were from Uttarakhand. Approximately 42.6% of the patients or their attendants were from Uttar Pradesh. Only 2.1% of the patients who approached telemedicine OPD were from other states of India (Table 2).

**Table 2:** Geographical distribution of patients availing teleconsultation at the virtual OPD (N=5,278) OPD, outpatient department

State	Number (%)
Uttarakhand	2918 (55.3%)
Uttar Pradesh	2246 (42.6%)
Others	114 (2.1%)

Around 17.6% of the teleconsultations were conducted for radiotherapy. General medicine and cardiology consultations were 15.2% and 12.8%, respectively. Approximately 7.7% of the calls of patients were required for orthopedic consultation. For Chemotherapy, General Surgery, Pediatrics, and obstetrics and gynecology (OBG), 6.5%, 5.1%, 4.5%, and 3.7% of the calls were attended, respectively (Table 3).

**Table 3:** Distribution of patients availing teleconsultation at the virtual OPD according to various specialties (N=5,278) CTVS, cardiothoracic and vascular surgery; OPD, outpatient department; PMR, physical medicine and rehabilitation

Clinical specialties	Number (%)
General medicine	801 (15.2%)
General surgery	271 (5.1%)
Orthopedics	407 (7.7%)
Pediatrics	240 (4.5%)
Obstetrics and gynecology	196 (3.7%)
Dermatology	77 (1.5%)
Psychiatry	93 (1.8%)
Radiology	10 (0.2%)
Ophthalmology	71(1.3%)
ENT	93(1.8%)
Community and family medicine	134 (2.5%)
Hematology	22 (0.4%)
Medical oncology	345 (6.5%)
Surgical oncology	64 (1.2%)
Gastroenterology	103 (2%)
Plastic surgery	20 (0.4%)
Neurosurgery	61 (1.2%)
Neurology	123 (2.3%)
Radiotherapy	930 (17.6%)
Urology	209 (4%)
Nephrology	69 (1.3%)
Emergency medicine	28 (0.5%)
AYUSH	3 (0.1%)
Pathology	13 (0.2%)
Cardiology	676 (12.8%)
Pediatric surgery	8 (0.2%)
Pulmonary medicine	74 (1.4%)
Hemato-oncology	26 (0.5%)
PMR	2 (0.0%)
CTVS	46 (0.9%)
Breast clinic	50 (1%)
Dentistry	13 (0.2%)

Nearly three-fourths (68.7%) of the patients were counselled during telephonic consultation. Approximately 12.7% of the patients or their relatives were counselled, and medicines were prescribed. Medications were prescribed to 5% of the patients. A total of 11.1% of the patients were advised to undergo investigations. Only a very few patients (0.6%) were referred (Table 4).

**Table 4:** Details of action taken by the attending doctors for the patients availing teleconsultation at the virtual OPD (N=5,278) OPD, outpatient department

Variable	Number (%)
Counselling	3626 (68.7%)
Medicine prescribed	264 (5%)
Both counselling and medicines	672 (12.7%)
Investigation advised	588 (11.1%)
Counselling, medicine, and investigations advised	99 (1.9%)
Counselling and referred	29 (0.6%)

SWOT analysis of telemedicine consultation

Strengths

· The patients require no prior registration, and the consent is considered implied.

· Considering the pandemic, no physical contact with patients minimizes the risk of exposure as consultation is through telecommunication.

· There is reduced out-of-pocket expenditure as traveling and other expenses are excluded.

· Human resources are better utilized.

· Accessibility of primary and tertiary healthcare through Teleconsultation is made to all.

· Patients are comfortable with Teleconsultation with the present digitalized trends.

· Minimum infrastructure is required to practice telemedicine.

 Weaknesses

· Teleconsultation quality depends on the speed and connectivity of internet services.

· Minimal emergency services are provided by telemedicine consultation.

· For cases that require patient counselling or where there is a need for “breaking bad news,” telemedicine consultation is inappropriate.

· There is poor quality of report exchange.

· Services of telemedicine are inaccessible to patients who are illiterate or do not have access to technology.

· Often calls are made only for inquiry purposes rather than for consultation

· Arranging referral and transport services for interstate patients is difficult.

· The quality of patient data achieved is poor.

· There is an absence of physical examination of patients.

· There is a shortage of trained staff.

· The same doctor may not be available for follow-up.

 Opportunities

· The referral mechanism can be strengthened through telemedicine.

· For better patient diagnosis, point-of-care technology can be utilized through the hub-and-spoke model.

· Patient’s records can be digitalized through the creation of Electronic Health Record (EHR) and linking them to their Unique Health Identification Number (UHID).

· Geolocation of the patient can be used for an early investigation of a potential outbreak.

· Stringent guidelines should be proposed to avoid medicolegal issues and violation of privacy.

 Threats

· Record keeping is a major lacuna of teleconsultation, which may lead to medicolegal issues.

· The same physician may not be available for every teleconsultation.

· Privacy of the patient, especially vulnerable groups, while communicating with a doctor can be hampered.

· Poor quality of telecommunication due to inadequate internet speed or network issues may lead to poor quality patient care.

· Lack of awareness among beneficiaries regarding services being provided through telemedicine.

## Discussion

Sharing of symptoms and eliciting signs is crucial for the diagnosis of any health condition. There are a number of technologies that can be used in telemedicine, which can help patients adhere better to their medication regimens and manage their diseases better [2].

Alharbi et al. found that 54% of the patients were male and the remaining 46% were female [4]. Polinski et al. reported 70% of female patients in their study [5]. In comparison to this, in the present study, the proportion of males was 54.4% compared with female patients (45.6%) in the telemedicine OPD. This finding might be due to the many patients included in this study, different sex ratios of the population, and a study conducted for a longer duration. In this study, around 17.6% of the teleconsultations were performed for Radiotherapy. General medicine and cardiology consultations were 15% and 12.7%, respectively. Approximately 7.6% and 6.5% of patients' calls were for orthopedic consultation and chemotherapy, respectively. In contrast, Alharbi et al. in their study found that most (58.1%) of the participants attended a virtual clinic of family medicine and the dermatology and pediatrics virtual clinics were attended by 15% 11.8% of the participants, respectively [4].

Telemedicine offers a variety of healthcare services by the healthcare providers, especially by the doctors, through the virtual clinic. In our study, the modes of communication were video, audio, and text messages. Telemedicine was instrumental during the pandemic when the lockdown was enforced worldwide. In Paris, among all the general practitioners, approximately 44% of them attended at least one teleconsultation during a pandemic [6]. With the improvement in communication technologies, telemedicine depicts a succession of medicine, and it should be established in all urban medical centers [7]. During the COVID-19 pandemic, virtual OPDs of AIIMS Rishikesh provided healthcare facilities to thousands of patients from all over India, especially during the lockdown period when patients had no option other than virtual consultation with the doctors/consultants. Internet-based audio and video services were also offered through WhatsApp.

Telemedicine is cost-effective because of the falling cost of internet services and cheaper devices [8]. During the COVID-19 pandemic, telemedicine services act as a barrier to the spread of COVID infection [9]. Telemedicine comes up with 4Cs: “care, convenience, comfort, and confidentiality” [10,11]. Telemedicine services save the important time and money of those patients who are critically ill and need urgent emergency care. Triage could be faster and more efficient in an emergency by implementing telemedicine in emergency care and helps in improvements in waiting time and patient satisfaction [12].

During the lockdown period in India, when there was no opportunity for a face-to-face consultation with the doctor for chronic diseases, and most patients benefitted from telemedicine at AIIMS Rishikesh. Telemedicine was a useful tool to manage patients with diabetes during the lockdown period [13]. This study was conducted to assess the SWOT and profile of patients attending virtual OPD under telemedicine at AIIMS Rishikesh. Telemedicine's main benefit is that it allows patients to communicate with doctors while at home and thus helps the patients avoid the risk of getting the infection by coronavirus as avoiding the visit to the hospital for the treatment [14]. The disadvantage of telemedicine could be the clinical diagnosis errors because of the lack of examination of the patients. The diagnosis is completely dependent on the patients' history and/or their investigation reports, which are also difficult to obtain during the lockdown period due to the COVID-19 pandemic.

Professionalism should be maintained throughout the teleconsultation/virtual meeting with patients by using a high-resolution camera and seeing their face in the video. There is a need for a high-quality Wi‐Fi or network signal [15].

In this study, around 22% of the patients who approached virtual OPD were ≥60 years of age. In comparison, Alharbi et al. in their study including 439 respondents found that 12.3% of the patients were ≥ 60 years of age [4]. This finding might be due to the demographic composition of the population, and patients more than 60 years of age had a higher chance of getting a severe infection by COVID-19, and travel is also risky during the COVID-19 pandemic. Consultation utilizing Telemedicine was more favored by the patients aged 68 years (range: 58-75 years) than patients aged 76 years (range: 70-79.2 years) during the COVID-19 pandemic [16]. During the pandemic, telemedicine proved to be a reliable technique not only for patient consultation but also for follow-up, as clinically unnecessary visits may increase COVID-19 exposure and infectious risk for patients and providers [17].

In the present study, more than half (55.3%) of the patients or their attendants who consulted telemedicine OPD were from Uttarakhand. Approximately 42.6% and 2.1% of the patients or their attendants who approached telemedicine OPD were from Uttar Pradesh and other states of India, respectively. In this study, around two-thirds (68.7%) of the patients were counselled during telephonic consultation. Approximately 12.7% of the patients or their relatives were counselled, and medicines were prescribed. Medications were prescribed to 5% of patients. A total of 11.1% of the patients were advised to undergo investigations. Telemedicine has become a critical tool for protecting access to care and responding to public health demands as a result of the COVID-19 pandemic. However, it has stressed the importance of addressing concerns about a lack of access to technology and high-speed internet, as well as inadequate digital literacy, which may hinder some populations from obtaining virtual treatment [18].

## Conclusions

In this study, we performed SWOT analysis of telemedicine consultations. Telemedicine can be effective if certain requirements had been provided. Record keeping is a major lacuna of teleconsultation. Around 22% of the patients who approached telemedicine OPD were ≥ 60 years of age. The proportion of male patients was higher than that of female. More than half of the patients or their attendants who consulted telemedicine OPD were from Uttarakhand. Maximum consultations were performed for radiotherapy, and most of the patients were counselled during teleconsultation.
